# Metabolomics analysis of *Pseudomonas chlororaphis* JK12 algicidal activity under aerobic and micro-aerobic culture condition

**DOI:** 10.1186/s13568-018-0660-x

**Published:** 2018-08-20

**Authors:** Jaejung Kim, Xiao Mei Lyu, Jaslyn Jie Lin Lee, Guili Zhao, Seow Fong Chin, Liang Yang, Wei Ning Chen

**Affiliations:** 1 School of Chemical and Biomedical Engineering, College of Engineering, Nanyang Technological University, 62 Nanyang Drive, Singapore, 637459 Singapore; 2 Singapore Centre for Environmental Life Sciences Engineering, Nanyang Technological University, 60 Nanyang Drive, Singapore, 637551 Singapore

**Keywords:** Algicidal bacteria, *Pseudomonas chlororaphis*, Culture condition, Aerobic, Micro-aerobic, Metabolomics

## Abstract

Utilization of algicidal bacteria as a biological agent have been receiving significant interest for controlling harmful algal blooms. While various algicidal bacterial strains have been identified, limited studies have explored the influence of bacterial culture conditions on its algicidal activity. Here, the effect of oxygen on the algicidal activity of a novel bacterium JK12, against a model diatom, *Phaeodactylum tricornutum* (*P. tricornutum*) was studied. Strain JK12 showed high algicidal activity against *P. tricornutum* and was identified as *Pseudomonas chlororaphis* (*P. chlororaphis*) by 16S ribosomal RNA gene analysis. JK12 culture supernatant exhibited strong algicidal activity while washed JK12 cells showed no obvious activity, indicating that JK12 indirectly attacks algae by secreting extracellular algicidal metabolites. Micro-aerobic culture condition dramatically enhanced the algicidal activity of JK12 by 50%, compared to that cultured under aerobic condition in 24 h. Extracellular metabolomic profiling of JK12 using gas chromatography–mass spectrometry and liquid chromatography–mass spectrometry analysis revealed significantly higher amounts of allantoic acid, urocanic acid, cytidine 2′,3′-cyclic phosphate, uridine 2′,3′-cyclic phosphate, and chlorinated tryptophan in the micro-aerobic culture. This is the first report to demonstrate the important role of oxygen on the algicidal activity of a non-pathogenic strain *P. chlororaphis*. In addition, the metabolomics analysis provided insights into the algicidal mechanism of *P. chlororaphis*.

**Electronic supplementary material:**

The online version of this article (10.1186/s13568-018-0660-x) contains supplementary material, which is available to authorized users.

## Introduction

Harmful algal blooms (HABs), is a phenomenon that describes the uncontrolled proliferation of certain algal species, which have been increasingly occurring across the world (Hallegraeff 1993). HABs causes harm either through toxin release or high algae biomass (GEOHAB 2001). The former could lead to human seafood poisoning while the latter could result in oxygen depletion in the water, causing massive mortality of aquatic organisms, and altering the marine ecosystem. The cause behind frequent outbreaks of HABs, could be due to excessive supply of nutrients, particularly phosphorus and nitrogen, from human activities, which leads to nutrient enrichment and favorable bloom condition for algae growth (Anderson et al. 2002; Heisler et al. 2008).

Various strategies have been developed to control HABs, ranging from mechanical control such as deposition of clay forms (Na et al. 1996), to chemical control such as toxic chemical release (Rounsefell and Evans 1958), and environmental manipulation such as physical or chemical modifications of the environment (Anderson 2009). However, these methods have the disadvantage which includes obscure cost/benefit rationale, poor species-specificity, and possible detrimental effects to the phytoplankton community. Recently, biological control to tackle HABs has been attracting significant attention, especially the use of bacteria as an algicidal agent (Cho 2012; Jung et al. 2012; Kang et al. 2011; Oh et al. 2011; Yu et al. 2018).

Bacteria have been closely associated to termination of harmful algal bloom by inhibiting or killing the algae (Mayali and Azam 2004). Several algicidal bacteria have been identified to date, including *Pseudomonas* spp. (Wang et al. 2005), *Vibrio* spp. (Li et al. 2014), *Alteromonas* spp. (Oh et al. 2011), and *Bacillus* spp. (Zhao et al. 2014). In general, algicidal bacteria attacks algae by employing a direct or indirect approach (Skerratt et al. 2002). Direct approach involves physical contact with algae cells, while the indirect approach utilizes secreted algicidal metabolites which lyses the algae.

To enhance the algicidal efficacy of isolated bacteria, several environmental parameters that affect the susceptibility of algae to algicidal bacteria have been studied. One study showed that the culture temperature and algal density of *Microcystis aeruginosa* affected its susceptibility to algicidal bacteria (Su et al. 2016). Another study reported a linear relationship between algicidal efficiency and the growth rate of *M. aeruginosa*, and suggested environmental conditions influencing the growth of *M. aeruginosa* could impact the efficacy of algicidal bacteria (Shao et al. 2015). Although the approaches showed some improvement in algicidal efficacy, the investigated environmental parameters suffer inherent limitations for practical applications because parameters such as temperature in the lake or ocean is difficult to be controlled or readily manipulated. As such, it is important to identify conditions that can be precisely controlled and altered to increase algicidal efficacy.

Several diatoms form algal blooms and dominate the phytoplankton community (D’Alelio et al. 2010; Kang et al. 2011; Paul and Pohnert 2011; Ruggiero et al. 2017). *Phaeodactylum tricornutum* (*P. tricornutum*) is a marine microalgae species that belongs to the diatom group. In addition, *P. tricornutum* is one of the two diatoms that is completely genome sequenced (Bowler et al. 2008), positioning it as a model diatom for studies. As such, we evaluated the algicidal activity of the bacterium JK12 strain against *P. tricornutum* and identified the species most related to JK12 using 16s ribosomal RNA (rRNA) gene sequence analysis. We determined the algicidal mode of JK12 and investigated the algicidal activity of JK12 in response to different oxygen availability during fermentation. The extracellular metabolic profile of the JK12 filtrate from aerobic and micro-aerobic culture were analyzed and compared by using gas chromatography–mass spectrometry (GC–MS) liquid chromatography–mass spectrometry (LC–MS) analysis.

## Materials and methods

### Algae culture

*Phaeodactylum tricornutum* UTEX LB 642 was ordered from Culture Collection of Algae at The University of Texas at Austin (UTEX), USA. *P. tricornutum* were grown at 20 ± 1 °C under light intensity of 50 μmol photons m^−2^ s^−1^ in f/2 + silica medium (Guillard 1975) prepared with 0.22-μm filtered natural sea water supplied by Tropical Marine Science Institute (TMSI), Singapore.

### Bacteria culture

Bacterial strain JK12 was identified from a local strain collection from Dr. Liang Yang, Nanyang Technological University, Singapore. This strain has been deposited in the NCIMB under accession number NCIMB 15121. Strain JK12 was cultured in Luria–Bertani (LB) broth at 30 °C under shaking at 200 rpm under aerobic or micro-aerobic conditions. Aerobic culture condition was made by inoculating JK12 in 50 mL LB in a 250 mL Erlenmeyer flask with a vent cap while micro-aerobic culture condition was made by inoculating JK12 in 5 mL LB in a 50 mL falcon tube with tightly closed cap.

### Evaluation of algicidal activity and algicidal mode

To determine the algicidal activity of strain JK12 against *P. tricornutum*, strain JK12 was cultured for 24 h under aerobic condition to reach stationary phase. Subsequently, 100 μL of JK12 culture was added into 900 μL of exponentially growing *P. tricornutum* culture in a 24-microwell plate. The control group comprised equal volume of sterile f/2 medium added into algal culture to ensure consistent total volume. The plate was incubated in algae culturing condition for 24 h and then the fluorescence intensity of treated groups and control groups were measured using a microplate reader with 440 nm of excitation light and 680 nm of emission light. The intensity of fluorescence emission is proportional to the amount of in vivo chlorophyll-a for the estimation of algal biomass (Andersen 2005; Lorenzen 1967; Richards and Thompson 1952). The algicidal rate was calculated using the following formula:

$$Algicidal\,rate\,(\%) = \frac{FC - FT}{FC}\, \times 100$$

where FT is the fluorescence intensity of the treated algal culture and FC is the fluorescence intensity of the control algal culture (Li et al. 2016; Yu et al. 2018; Zhang et al. 2014).

The algicidal mode was evaluated by applying different experimental conditions: (1) 10% v/v of bacteria culture (2) 10% v/v of 0.22-μm Millipore membrane filtered bacterial culture supernatant after centrifugation of the JK12 culture at 5000×*g* for 10 min (3) 10% v/v of washed JK12 cells after centrifugation of the bacterial culture at 5000×*g* for 10 min followed by washing twice and re-suspending in f/2 + silica medium (4) 10% v/v of the sterile LB. Control group consisted of 10% v/v of f/2 + silica medium in algal culture. Mixture was incubating under algae culturing condition for 24 h and the algicidal rate was evaluated as described above.

### Characterization of the active algicidal substance

Sensitivity of the algicidal substance to heat was tested by incubating culture supernatant prepared after 24 h of JK12 growth to 95 °C for 15 min. After which, the treated supernatant was added into algal culture at a volume fraction of 10.0% (v/v) for 24 h followed by examination of algicidal rate.

The algicidal substance was also autoclaved at 121 °C for 15 min under 15 psi to test its susceptibility to combination of high heat and pressure. After which, the autoclaved supernatant was added into algal culture at a volume fraction of 10.0% (v/v) for 24 h followed by examination of algicidal rate.

### Identification of the JK12

Genomic DNA of the strain JK12 was purified according to manufacturer’s instruction using QIAamp DNA Mini Kit (Qiagen). The concentration, purity and integrity of DNA was checked using quantitative spectrophotometric assay and electrophoresis (1% agar). The genome was then sequenced using Illumina HiSeq 2000 platform and de novo assembly was performed using CLC Genomics workbench 10.0 (Qiagen). The 16S rRNA sequence (GenBank accession number MH 322032) was then annotated using rapid annotation using subsystem technology (RAST) (Aziz et al. 2008) and compared with other bacterial 16S rRNA sequences obtained from the GenBank database (http://www.ncbi.nlm.nih.gov/blast) using the BLAST program. Phylogenetic tree was then constructed using MEGA 7.0 by neighbor-joining method.

### Examining the influence of oxygen on JK12 growth

To study the effect of oxygen on JK12 growth, bacteria was grown under aerobic or micro-aerobic condition, as described above. The growth of JK12 was measured at different time-points (4, 8, 12, 24, 48 h) using a spectrophotometer with OD_600_. The starting OD_600_ of JK12 was fixed to 0.01.

### Examining the influence of oxygen on algicidal activity by JK12

JK12 was grown under aerobic or micro-aerobic conditions as described above to investigate the influence of oxygen on its algicidal activity. JK12 culture supernatant was extracted at different time-points (8, 12, 24, 48 h) that reflects different growth stages of JK12. Subsequently, 10% v/v of the extracted culture supernatant was added into an exponentially growing algal culture. The mixture was incubated under algae culturing condition for 24 h and the algicidal rate was examined as described above.

### GC–MS analysis of JK12 culture supernatant

JK12 was grown for 24 h under aerobic and micro-aerobic condition and 400 μL of JK12 culture supernatant was mixed with 10 μL of ribitol (Sigma-Aldrich, St. Louis MO, USA), which served as an internal standard. The samples were then freeze-dried to concentrate the metabolites. Methoximation was carried out by dissolving the lyophilized samples in 50 μL of 20 mg/mL methoxyamine hydrochloride dissolved in pyridine and incubating at 37 °C for 1 h. Subsequently, silylation was performed by adding 100 μL of *N*-methyl-*N*-(trimethylsilyl)-trifluoroacetamide (MSTFA) with 1% trimethylchlorosilane (TMCS) to the samples and incubating at 70 °C for 30 min. Afterwards, the samples were vortexed for 60 min at room temperature and analyzed in GC–MS. GC-MS system (Agilent Technologies 7890A-5975C) was equipped with HP-5MS column (30 m × 250 μm × 0.25 μm Agilent J&W Scientific, Folsom, CA, USA). 1 µL of sample volume was injected in split less mode. The injector temperature and ion source temperature were set at 250 and 230 °C, respectively. The oven temperature was kept at 75 °C for 4 min, and was raised to 280 °C by 4 °C/min, and held for 2 min. Data were recorded from 35 to 600 *m*/*z* with a scan time of 0.2 s (Wang et al. 2010). Metabolites were identified by using the NIST08 mass spectral library. Samples were normalized using the internal standard, ribitol.

### LC–MS analysis of JK12 culture supernatant

JK12 was grown for 12 h under aerobic and micro-aerobic condition and the culture supernatant was collected and transferred to glass vial for LC–MS analysis. Ultra-high-performance liquid chromatography (UPLC) system coupled with Xevo G2-XS quadrupole-time-of-flight (Q-TOF) mass spectrometry (Waters Corp., USA) and electrospray ionization (ESI) were conducted for untargeted metabolome analysis. HSS T3 (1.8 µm; 2.1 × 100 mm) column was used and the column temperature was set at 45 °C and the auto sampler at 4 °C. Mobile phase A comprised 0.1% formic acid in water and mobile phase B comprised 0.1% formic acid in acetonitrile. An injection volume of 15 µL was used for both positive and negative ionization polarity modes. Collected data were centroided and analyzed using Progenesis QI software (Waters Corp., USA). The peaks were normalized to internal standards, hippuric acid-D5 and l-phenylalanine-^13^C9, ^15^N and subsequently filtered based on ANOVA p-value < 0.001.

## Results

### Algicidal activity and algicidal mode of strain JK12

**Fig. 1:**
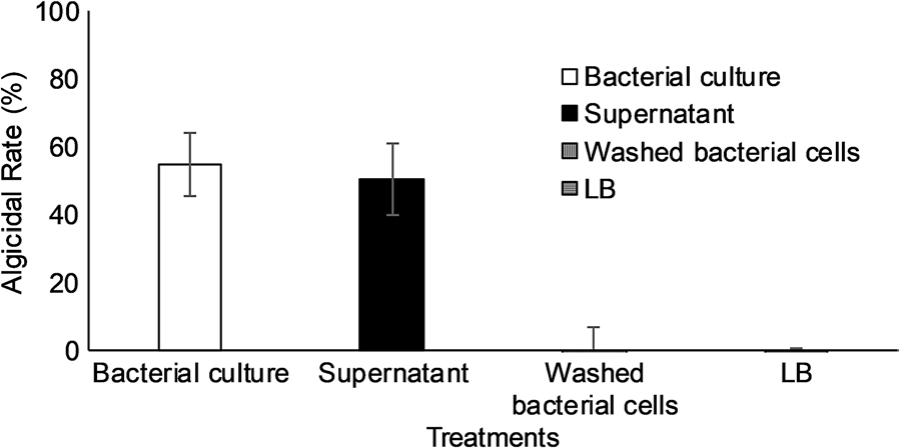
Evaluation of algicidal mode of JK12. All error bars indicate the SD of the three biological replicates

As shown in Fig. 1, strain JK12 cultured under aerobic condition for 24 h, exhibited high algicidal activity (55%) against *P. tricornutum* culture in 1 day. The algicidal mode of strain JK12 was determined by comparing the algicidal activity between the bacterial culture, cell-free filtrate, and washed JK12 cells and sterile LB broth against the algal culture. Bacterial culture of strain JK12 and the cell-free filtrate exhibited significant lysis of algae, at 55% and 51%, respectively, as compared to the control group. On the other hand, washed bacterial cells and sterile LB broth exhibited negligible algicidal activity.

### Stability of algicidal compounds

**Fig. 2:**
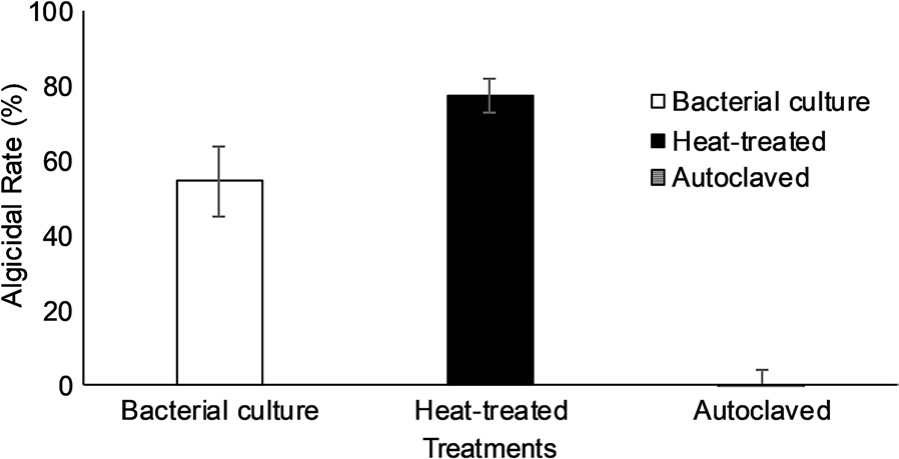
Characterization of the algicidal compounds. All error bars indicate the SD of the three biological replicates

Algicidal activity of KJ culture supernatant after heat treatment were determined as shown in Fig. 2. The heat-treated supernatant showed higher algicidal activity (77%) than intact supernatant (55%). However, when the supernatant was autoclaved, no algicidal activity was observed.

### Identification of JK12

**Fig. 3:**
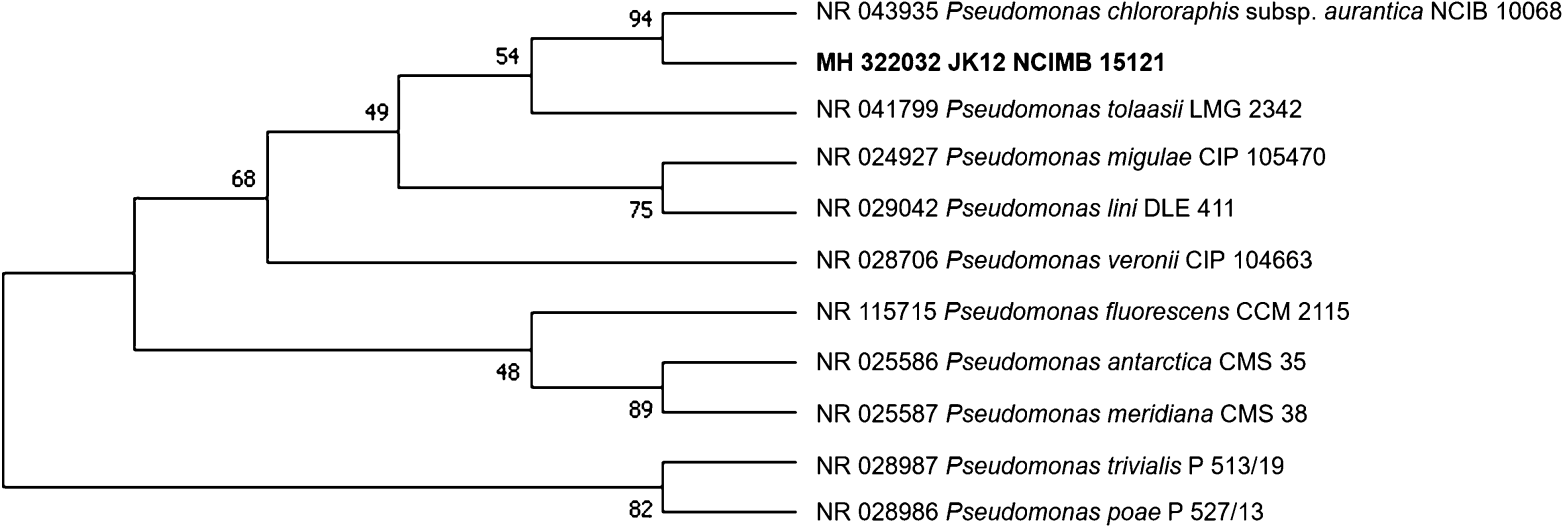
Phylogenetic tree of JK12 based on 16 s rRNA sequences obtained from GenBank

Constructed phylogenetic tree based on 16S rRNA gene sequence (GenBank accession number MH322032) analysis using MEGA software showed that 16S rRNA sequence of strain JK12 was in the closest relationship to the species *Pseudomonas chlororaphi*s (*P. chlororaphis*) as shown in Fig. 3. As such, strain JK12 was designated *P. chlororaphis*.

### Influence of oxygen on JK12 growth

**Fig. 4:**
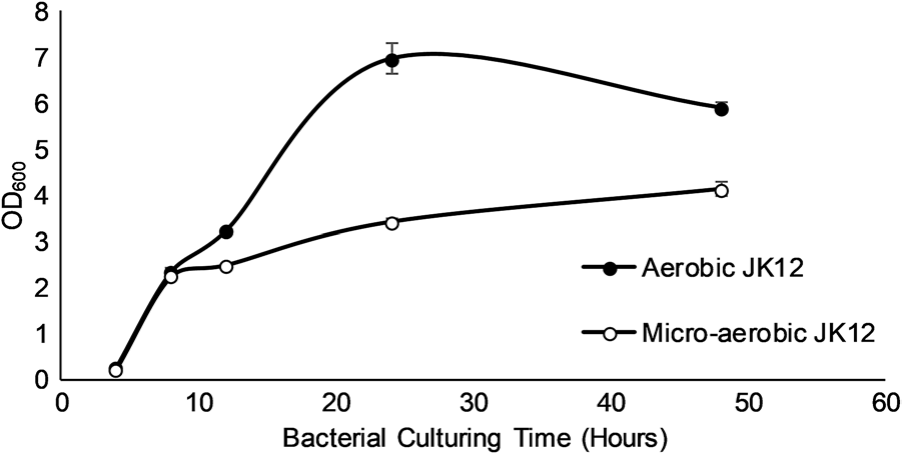
Effects of the oxygen on the growth of JK12. All error bars indicate the SD of the three biological replicates

OD_600_ of strain JK12 cultured under microaerobic or aerobic condition was measured at different time-points (8, 12, 24, and 48 h) to study the effect of oxygen on JK12 growth (Fig. 4). Under aerobic condition, JK12 entered exponential phase in 8 h followed by stationary phase in 24 h and eventually reached death phase at 48 h. On the other hand, the growth of JK12 under microaerobic condition was hindered from 8 h onwards. As a result, OD_600_ of micro-aerobic culture was approximately half of that of aerobic culture in 24 h.

### Influence of oxygen on algicidal activity by JK12

**Fig. 5:**
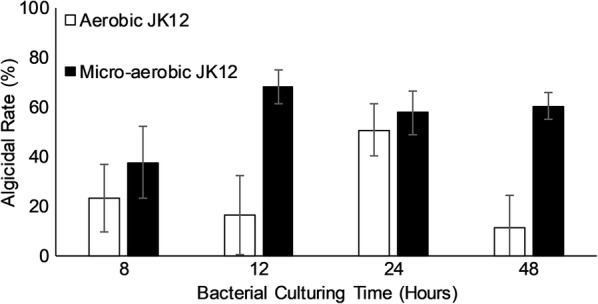
Effects of the bacterial supernatant on algae death rate of *P. tricornutum* at different time-points. All error bars indicate the SD of the three biological replicates

To study the influence of oxygen on algicidal activity by JK12, culture supernatant of strain JK12 cultured under microaerobic or aerobic condition were added into algal cultures at different time-points (8, 12, 24, and 48 h) that corresponds to different growth stages (early exponential phase, mid-exponential phase, stationary phase, and death phase) of JK12 (Fig. 5). The amount of oxygen available to JK12 during culture, seemed to play a big role on the algicidal activity of JK12. When JK12 was grown under aerobic condition it exhibited high algicidal activity of 50% during stationary phase (24 h), while the other growth phases exhibited poor algicidal activity. On the other hand, JK12 grown under microaerobic condition exhibited high algicidal activity (> 50%) from 12 h onwards of growth and maintained high algicidal activity throughout the measured experimental time-points (24 h and 48 h).

### Metabolite profiling of aerobic and micro-aerobic culture supernatant of strain JK12 using GC–MS

**Fig. 6:**
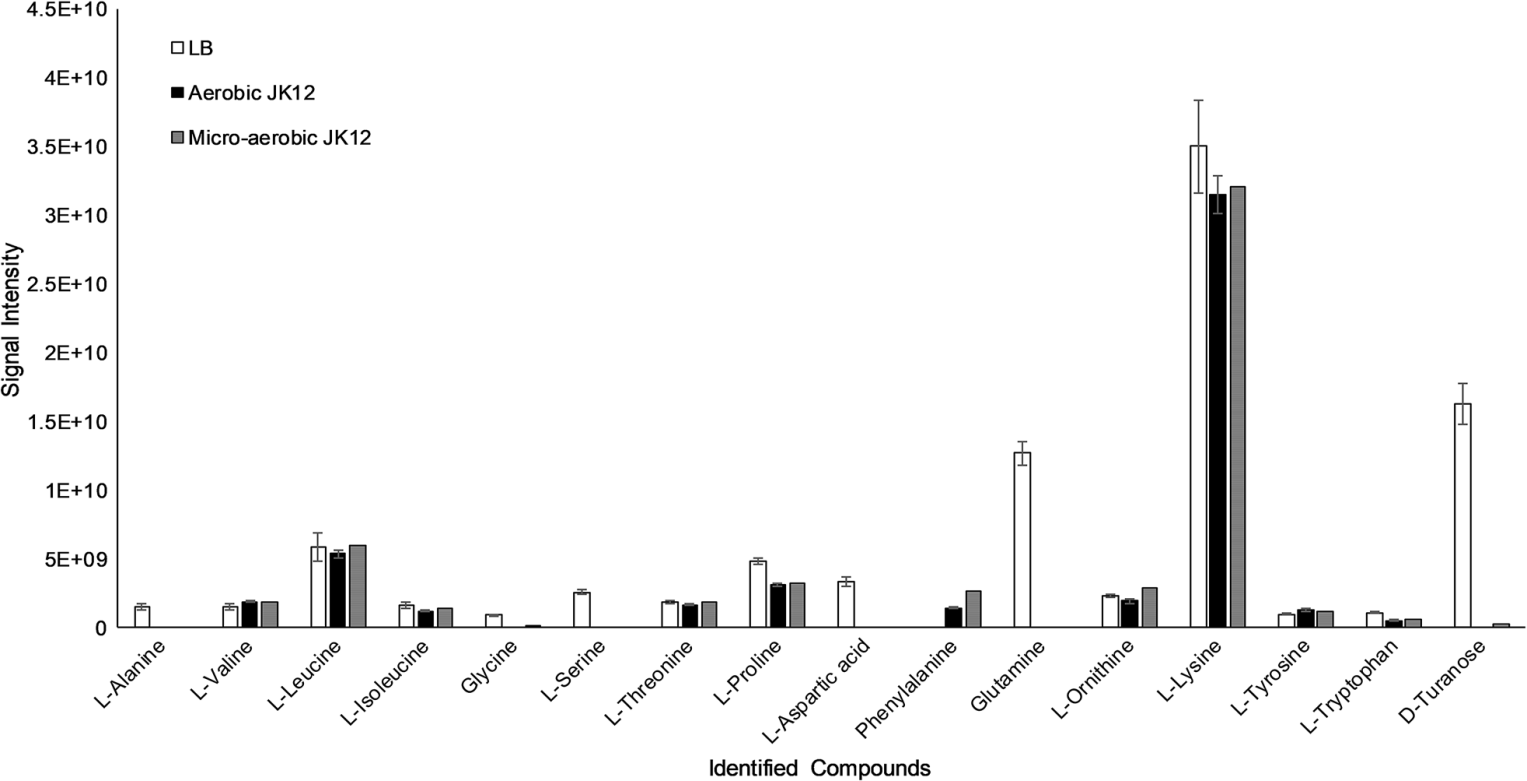
Metabolic profiling of the aerobic and micro-aerobic culture supernatant of JK12. Sterile LB was used as a control group. All error bars indicate the SD of the three biological replicates

**Fig. 7:**
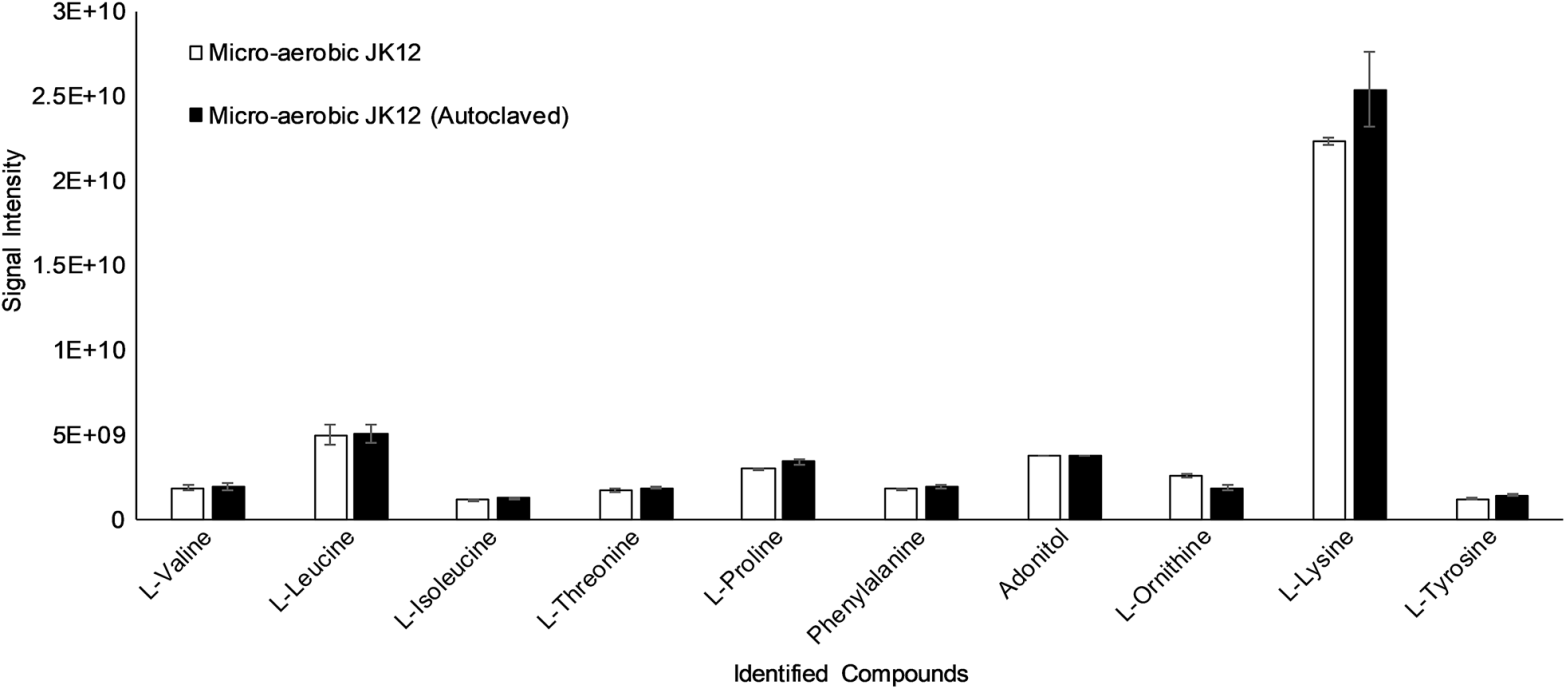
Metabolic profiling of autoclaved micro-aerobic culture supernatant of JK12. All error bars indicate the SD of the three biological replicates

To investigate the extracellular metabolomic profile of strain JK12 grown under aerobic and microaerobic conditions, culture supernatant of strain JK12 was analyzed by GC–MS. Aerobic and micro-aerobic cultures of JK12 did not show major differences in the number of identified metabolites. However, substantial differences were observed in the amount of each identified metabolites (Fig. 6). Sterile LB broth as a control generally showed the greatest amount of amino acids followed by micro-aerobic culture supernatant of JK12 and aerobic culture supernatant. Interestingly, no obvious differences in the metabolomic profile were observed before and after autoclaving the micro-aerobic culture supernatant of JK12 (Fig. 7).

### Metabolite profiling of aerobic and micro-aerobic culture supernatant of strain JK12 using LC–MS

ESI mass spectrum recorded by LC–MS analysis in positive ion mode revealed remarkable difference in the extracellular metabolomic profile of strain JK12 in aerobic (Additional file 1: Fig. S1a) and micro-aerobic (Additional file 1: Fig. S1b) conditions. Similar differences were observed in ESI mass spectrum recorded LC–MS analysis in negative ion mode (Additional file 1: Fig. S2a, b). In addition, principal component analysis plot based on LC–MS analysis in both ionization modes (Additional file 1: Figs. S3, S4) provided another evidence that clearly indicates the significant differences between the JK12 metabolites from two culture conditions. JK12 under micro-aerobic condition showed more amino acids compared to aerobic condition for both ionization modes (Additional file 1: Tables S1, S2), which was consistent to the GC–MS analysis results. Notably, micro-aerobic culture supernatant of JK12 contained significant amount of allantoic acid, urocanic acid, uric acid, cytidine 2′,3′-cyclic phosphate (cyclic CMP), uridine 2′,3′-cyclic phosphate (cyclic UMP), and chlorinated-tryptophan as shown in Tables 1 and 2. Heatmap correlation of JK12 metabolites visualized such differences between the two media as shown in Additional file 1: Figs. S5, S6.

**Table 1:** Shortlisted extracellular metabolites showing significant fold change in micro-aerobic culture compared to aerobic culture by LC–MS analysis in positive ionization mode

Identified molecule	Fold change
Allantoic acid	58.69
Cytidine 2′,3′-cyclic phosphate	14.53
Urocanic acid	22.77
Uridine 2′,3′-cyclic phosphate	11.86
Uric acid	8.49
l-4-Chlorotryptophan	115.08

**Table 2:** Shortlisted extracellular metabolites showing significant fold change in micro-aerobic culture compared to aerobic culture by LC–MS analysis in negative ionization mode

Identified molecule	Fold change
Cytidine 2′,3′-cyclic phosphate	14.26
Uridine 2′,3′-cyclic phosphate	16.15
Uric acid	8.59
l-4-Chlorotryptophan	405.43

## Discussion

*Pseudomonas chlororaphis* has been an organism of interest as an effective biocontrol agent, against various fungal pathogens in plants. This is due to the bacterium’s broad spectrum of antifungal activity, through the production of various active metabolites. This includes phenazine derivatives, hydrogen cyanide, and pyrrolnitrin (Chin-A-Woeng et al. 1998). In addition, *P. chlororaphis* is particularly attractive as a biocontrol agent, because it is generally regarded as being non-pathogenic to humans, wildlife or the environment (Anderson et al. 2018; Chen et al. 2015; Lee et al. 2011). This bacteria is also safe to use in agriculture, feed crops, and food (Anderson et al. 2018). Our study adds on to the attractive properties of *P. chlororaphis*, by demonstrating the first time that *P. chlororaphis* JK12 possesses high algicidal activity, which shows high potential as a bio-controller of harmful algal bloom.

The algicidal mode of JK12 was analyzed and the results in Fig. 1 showed that washed JK12 cells exhibited no obvious algicidal activity, whereas JK12 culture and culture supernatant exerted high algicidal activity, suggesting that JK12 employs an indirect algicidal mode, secreting active algicidal metabolites into the medium. In addition, algicidal activity remained high even after the culture supernatant was subjected to heat treatment, which indicated that the active algicidal metabolite, is most probably not a protein. Thus, further analysis on the culture supernatant was carried out without the use of proteomics.

We have discovered that the availability of oxygen during JK12 growth had a strong influence on the growth and algicidal activity of JK12 (Fig. 4). Under an oxygen deprived condition, the growth of JK12 was significantly suppressed. However, micro-aerobic culture condition imparted a superior algicidal activity to JK12 during all growth phases, achieving up to 51% increased algal lysis rate as compared to when it was cultured in aerobic condition (Fig. 5). Notably, micro-aerobic culture condition led to high algicidal activity especially from 12 h of growth onwards, which coincided with the onset of oxidative stress and inhibited growth of JK12. On the contrary, JK12 cultured under aerobic condition showed high algicidal activity only during stationary phase, despite showing a much higher growth as compared to that under micro-aerobic condition. This suggested that the bacterium could have adopted an alternative metabolic mode under oxygen limitation, leading to higher production of algicidal metabolites which far outweighed the drawback of reduced cell yield, which ultimately resulted in dramatically enhanced algicidal activity. Many studies have reported the significant effect of oxygen availability, on the biochemical properties of bacteria (McCloskey et al. 2014; Partridge et al. 2007; Portela et al. 2014; Shan et al. 2012; Trotter et al. 2011). Aerobic respiration is conventionally the favored metabolic mode as compared to microaerobic respiration. Such preference for a particular metabolic mode is based on energy conservation efficiency (Guest et al. 1996). Aerobic respiration offers the most efficient energy conservation metabolic mode as it enables complete oxidation of the substrate, generating the most number of adenosine triphosphate (ATP) from commonly used substrates compared to microaerobic respiration and fermentation (Guest et al. 1996). On the other hand, bacteria needs to undergo major adjustments at the metabolome level to adapt under oxygen limited conditions (Trotter et al. 2011).

Clear differences between aerobic and microaerobic cultures in relation to extracellular metabolomic profile were observed (Additional file 1: Figs. S1–S6). While both growth cultures showed various types of natural amino acids, greater amount of amino acids were present in micro-aerobic culture. Several amino acids have been previously reported to exhibit algicidal activity. For example, l-lysine secreted by *Streptomyces phaeofaciens* S-9 was reported to lyse cyanobacterial cells (Yamamoto et al. 1998). l-Lysine has also shown algicidal activity against *Microsystis* cells (Hehmann et al. 2002). However, we decided to exclude natural amino acids as the potential active algicidal metabolite(s) of JK12. This was mainly because LB as a control showed no obvious algicidal activity, although it contained the highest amount of amino acids as compared to aerobic or micro-aerobic culture supernatant of JK12 (Fig. 7). The rich amount of amino acids detected in both cultures could be attributed to LB, since it was used as the culture medium for JK12. In addition, autoclaving micro-aerobic culture supernatant removed the algicidal activity but showed similar metabolic profile between the two micro-aerobic cultures—before and after autoclaving. This further supports that natural amino acids found in JK12 are not likely the active compound(s) responsible for the algicidal activity.

Excluding amino acids, the interesting metabolites were allantoic acid, urocanic acid, uric acid, cyclic CMP and cyclic UMP and chlorinated tryptophan, which were 58-, 22-, 8-, 14-, 11-, 115-fold more in micro-aerobic culture as compared to aerobic culture, respectively (Table 1). Reports have clearly shown the relationship between the bacteria metabolites and algicidal activity of bacteria by identifying the specific algicidal metabolite, and subsequently demonstrating the algicidal activity of the pure algicidal compound. For example, urocanic acid was identified to be one of the active metabolites of a bacteria that exhibited high algicidal activities against harmful algal species including *Phaeocystis globosa*, *Skeletonema costatum*, *Prorocentrum donghaiense* and *Heterosigma akashiwo* (Zhao et al. 2014). Given that micro-aerobic culture of JK12 contained rich amount of urocanic acid, this could be a potential candidate responsible for the algicidal efficacy of JK12. Allantoic acid and uric acid are also interesting metabolites as numerous organic acids have been identified to possess algicidal activity. For example, l-2-azetidinecarboxylic acid selectively inhibited the growth of a few algae species such as the red-tide microalgae *Cochlodinium polykrikoides* and blue–green algae *Microcystis aeruginosa* and *Anabaena affinis* (Kim et al. 2006). Another study showed the algicidal effect of 2,4-dichlorophenoxy acetic acid on *Cylindrospermum* spp. (Singh 1974). Also, 3-(3-indolyl)butanoic acid was reported to suppress the growth of green algae *Chlamydomonas* spp. (Nonomura et al. 2001). Chlorinated tryptophan could also be the active metabolite causing algal lysis as it showed a 405-fold change (Table 2) between culture mediums. Modified amino acids such as beta-cyanoalanine have been reported to exhibit algicidal activity against a few algae species such as *Microcystis aeruginosa* and *Microcystis viridis* (Yoshikawa et al. 2000). While such metabolites were previously reported to lyse algal cells, it was unexpected to observe substantial amount of cyclic CMP and cyclic UMP in the micro-aerobic culture supernatant of JK12. Further work is required to separate the JK12 metabolites, purify and eventually determine the most active bacterial metabolite(s) in killing algae.

While few studies have optimized bacteria culture conditions to enhance algicidal activity, only limited improvement were shown. For example, algal lysis rate was improved by 9.1% after optimizing the carbon source, nitrogen source, initial pH value, temperature, salinity, and rotational speed as well as the medium composition such as yeast extract, tryptone, soluble starch, MgSO_4_ and NaNO_3_ using a single-factor test method (Lin et al. 2013). Another study increased the algicidal ratio by 16.9% after optimizing the fermentation conditions including carbon source, nitrogen source, inoculum size, initial pH and fermentation time by combining uniform design with artificial neural network and genetic algorithm (Cai et al. 2014). Up to our knowledge, we demonstrated for the first time that depriving oxygen during bacterium growth could remarkably increase the algicidal activity by up to 50%, which is significantly higher than previous studies.

Some studies also investigated the algicidal mechanism of bacterial metabolites at the molecular level by measuring the transcription level of regulated genes in the affected algae cells. For instance, a study showed that *M. aeruginosa* cells treated with algicidal compound 3,4-dihydroxybenzalacetone (DBL) led to decreased expression in cell division gene *ftsZ* and peptidoglycan gene *glmS* of the algal cells, suggesting membrane damage as a target site for DBL (Jin et al. 2017). Another paper showed that *Phaeocystis globosa* treated with algicidal metabolites from bacteria expressed down-regulated transcriptional expression of photosynthesis-related genes such as *pbsA* and *rbcS*, indicating close association between photo inhibition and algicidal mechanism (Guan et al. 2015). Different algicidal mechanism may exist for different algicidal compounds and thorough investigation should be done to determine the mechanism of JK12 metabolites in lysing algal cells.

In conclusion, this is the first report to identify and demonstrate high algicidal activity of strain JK12 belonging to *P. chlororaphis*, which is generally considered as a non-pathogenic bacterium safe to the environment. Thus, *P. chlororaphis* JK12 could serve as an excellent biocontroller of HABs. Moreover, strain JK12 cultured under microaerobic condition exhibited superior algicidal activity across all growth stages compared to that grown under aerobic condition. This finding provides insight into exploiting oxygen level during bacterium growth to enhance algicidal activity and achieve greater control of harmful algal bloom.

## Additional file

**Additional file 1.** **Fig. S1**. ESI mass spectrum recorded by LC–MS analysis in positive ion mode for JK12 metabolites from **a** aerobic culture and **b** micro-aerobic culture. **Fig. S2**. ESI mass spectrum recorded by LC–MS analysis in negative ion mode for JK12 metabolites from **a** aerobic culture and **b** micro-aerobic culture. **Fig. S3**. PCA plot based on LC–MS analysis in positive ion mode for JK12 metabolites from aerobic and micro-aerobic culture. **Fig. S4**. PCA plot based on LC–MS analysis in negative ion mode for JK12 metabolites from aerobic and micro-aerobic culture. **Fig. S5**. Heatmap correlation based on LC–MS analysis in positive ion mode for JK12 metabolites from aerobic and micro-aerobic culture. **Fig. S6**. Heatmap correlation based on LC–MS analysis in negative ion mode for JK12 metabolites from aerobic and micro-aerobic culture. **Table S1**. List of identified metabolites extracted from JK12 media based on LC–MS analysis in positive ion mode. **Table S2**. List of identified metabolites extracted from JK12 media based on LC–MS analysis in negative ion mode.

## Abbreviations

HAB: harmful algal bloom
*P*. *tricornutum*: *Phaeodactylum tricornutum*
*P*. *chlororaphis*: *Pseudomonas chlororaphis*
rRNA: ribosomal RNA
GC–MS: gas chromatography–mass spectrometry
LC–MS: liquid chromatography–mass spectrometry
UTEX: University of Texas at Austin
TMSI: Tropical Marine Science Institute
LB: Luria–Bertani
RAST: rapid annotation using subsystem technology
MSTFA: *N*-methyl-*N*-(trimethylsilyl)-trifluoroacetamide
TCMS: trimethylchlorosilane
UPLC: ultra-high-performance liquid chromatography
Q-TOF: quadrupole-time-of-flight
ESI: electrospray ionization
CMP: cytidine 2′,3′-phosphate
UMP: uridine 2′,3′-phosphate
ATP: adenosine triphosphate
DBL: 3,4-dihydroxybenzalacetone

## References

[CR1] Andersen RA (2005). Algal culturing techniques.

[CR2] Anderson DM (2009). Approaches to monitoring, control and management of harmful algal blooms (HABs). Ocean Coast Manag.

[CR3] Anderson DM, Glibert PM, Burkholder JM (2002). Harmful algal blooms and eutrophication: nutrient sources, composition, and consequences. Estuaries.

[CR4] Anderson JA, Staley J, Challender M, Heuton J (2018). Safety of *Pseudomonas chlororaphis* as a gene source for genetically modified crops. Transgenic Res.

[CR5] Aziz RK, Bartels D, Best AA, DeJongh M, Disz T, Edwards RA, Formsma K, Gerdes S, Glass EM, Kubal M (2008). The RAST Server: rapid annotations using subsystems technology. BMC Genomics.

[CR6] Bowler C, Allen AE, Badger JH, Grimwood J, Jabbari K, Kuo A, Maheswari U, Martens C, Maumus F, Otillar RP (2008). The *Phaeodactylum* genome reveals the evolutionary history of diatom genomes. Nature.

[CR7] Cai G, Zheng W, Yang X, Zhang B, Zheng T (2014). Combination of uniform design with artificial neural network coupling genetic algorithm: an effective way to obtain high yield of biomass and algicidal compound of a novel HABs control actinomycete. Microb Cell Fact.

[CR8] Chen Y, Shen X, Peng H, Hu H, Wang W, Zhang X (2015). Comparative genomic analysis and phenazine production of *Pseudomonas chlororaphis*, a plant growth-promoting rhizobacterium. Genomics Data.

[CR9] Chin-A-Woeng TF, Bloemberg GV, van der Bij AJ, van der Drift KM, Schripsema J, Kroon B, Scheffer RJ, Keel C, Bakker PA, Tichy H-V (1998). Biocontrol by phenazine-1-carboxamide-producing *Pseudomonas chlororaphis* PCL1391 of tomato root rot caused by *Fusarium oxysporum f* sp. *radicis*-*lycopersici*. Mol Plant Microbe Interactions.

[CR10] Cho JY (2012). Algicidal activity of marine *Alteromonas* sp. KNS-16 and isolation of active compounds. Biosci Biotechnol Biochem.

[CR11] D’Alelio D, d’Alcala MR, Dubroca L, Zingone A, Montresor M (2010). The time for sex: a biennial life cycle in a marine planktonic diatom. Limnol Oceanogr.

[CR12] GEOHAB (2001) Global ecology and oceanography of harmful algal blooms, science plan. In: Glibert P, Pitcher G (eds). SCOR and IOC, Baltimore and Paris

[CR13] Guan C, Guo X, Li Y, Zhang H, Lei X, Cai G, Guo J, Yu Z, Zheng T (2015). Photoinhibition of *Phaeocystis globosa* resulting from oxidative stress induced by a marine algicidal bacterium *Bacillus* sp. LP-10. Sci Rep.

[CR14] Guest JR, Green J, Irvine AS, Spiro S (1996). The FNR modulon and FNR-regulated gene expression Regulation of gene expression in *Escherichia coli*.

[CR15] Guillard RR (1975). Culture of phytoplankton for feeding marine invertebrates culture of marine invertebrate animals.

[CR16] Hallegraeff GM (1993). A review of harmful algal blooms and their apparent global increase. Phycologia.

[CR17] Hehmann A, Kaya K, Watanabe MM (2002). Selective control of *Microcystis* using an amino acid–a laboratory assay. J Appl Phycol.

[CR18] Heisler J, Glibert P, Burkholder J, Anderson D, Cochlan W, Dennison W, Gobler C, Dortch Q, Heil C, Humphries E, Lewitus A, Magnien R, Marshall H, Sellner K, Stockwell D, Stoecker D, Suddleson M (2008). Eutrophication and harmful algal blooms: a scientific consensus. Harmful Algae.

[CR19] Jin P, Wang H, Liu W, Zhang S, Lin C, Zheng F, Miao W (2017). Bactericidal metabolites from *Phellinus noxius* HN-1 against *Microcystis aeruginosa*. Sci Rep.

[CR20] Jung SW, Kang Y-H, Baek SH, Lim D, Han M-S (2012). Biological control of *Stephanodiscus hantzschii* (Bacillariophyceae) blooms in a field mesocosm by the immobilized algicidal bacterium *Pseudomonas fluorescens* HYK0210-SK09. J Appl Phycol.

[CR21] Kang Y-H, Jung SW, Jo S-H, Han M-S (2011). Field assessment of the potential of algicidal bacteria against diatom blooms. Biocontrol Sci Tech.

[CR22] Kim J-S, Kim J-C, Lee S, Lee B-H, Cho KY (2006). Biological activity of l-2-azetidinecarboxylic acid, isolated from Polygonatum odoratum var. pluriflorum, against several algae. Aquat Bot.

[CR23] Lee JH, Ma KC, Ko SJ, Kang BR, Kim IS, Kim YC (2011). Nematicidal activity of a nonpathogenic biocontrol bacterium, *Pseudomonas chlororaphis* O6. Curr Microbiol.

[CR24] Li D, Zhang H, Fu L, An X, Zhang B, Li Y, Chen Z, Zheng W, Yi L, Zheng T (2014). A novel algicide: evidence of the effect of a fatty acid compound from the marine bacterium, *Vibrio* sp. BS02 on the harmful dinoflagellate, *Alexandrium tamarense*. PLoS ONE.

[CR25] Li Y, Lei X, Zhu H, Zhang H, Guan C, Chen Z, Zheng W, Fu L, Zheng T (2016). Chitinase producing bacteria with direct algicidal activity on marine diatoms. Sci Rep.

[CR26] Lin J, Zheng W, Tian Y, Wang G, Zheng T (2013). Optimization of culture conditions and medium composition for the marine algicidal bacterium *Alteromonas* sp. DH46 by uniform design. J Ocean Univ China.

[CR27] Lorenzen CJ (1967). Determination of chlorophyll and pheo-pigments: spectrophotometric equations 1. Limnol Oceanogr.

[CR28] Mayali X, Azam F (2004). Algicidal bacteria in the sea and their impact on algal blooms. J Eukaryot Microbiol.

[CR29] McCloskey D, Gangoiti JA, King ZA, Naviaux RK, Barshop BA, Palsson BO, Feist AM (2014). A model-driven quantitative metabolomics analysis of aerobic and anaerobic metabolism in *E. coli* K-12 MG1655 that is biochemically and thermodynamically consistent. Biotechnol Bioeng.

[CR30] Na G-H, Choi W-J, Chun Y-Y (1996). A study on red tide control with less suspension. J Aquacult.

[CR31] Nonomura T, Matsuda Y, Bingo M, Onishi M, Matsuda K, Harada S, Toyoda H (2001). Algicidal effect of 3-(3-indolyl) butanoic acid, a control agent of the bacterial wilt pathogen, *Ralstonia solanacearum*. Crop Protect.

[CR32] Oh J-I, Kim M-J, Lee J-Y, Ko I-J, Kim W, Kim SW (2011). Isolation and characterization of algicidal bacteria from *Cochlodinium polykrikoides* culture. Biotechnol Bioprocess Eng.

[CR33] Partridge JD, Sanguinetti G, Dibden DP, Roberts RE, Poole RK, Green J (2007). Transition of *Escherichia coli* from aerobic to micro-aerobic conditions involves fast and slow reacting regulatory components. J Biol Chem.

[CR34] Paul C, Pohnert G (2011). Interactions of the algicidal bacterium *Kordia* algicida with diatoms: regulated protease excretion for specific algal lysis. PLoS ONE.

[CR35] Portela CA, Smart KF, Tumanov S, Cook GM, Villas-Boas SG (2014). Global metabolic response of *Enterococcus faecalis* to oxygen. J Bacteriol.

[CR36] Richards FA, Thompson TG (1952). The estimation and characterization of plankton populations by pigment analyses. II. A spectrophotometric method for the estimation of plankton pigments. J Mar Res.

[CR37] Rounsefell GA, Evans JE (1958) Large-scale experimental test of copper sulfate as a control for the Florida red tide

[CR38] Ruggiero MV, D’Alelio D, Ferrante MI, Santoro M, Vitale L, Procaccini G, Montresor M (2017). Clonal expansion behind a marine diatom bloom. ISME J.

[CR39] Shan Y, Lai Y, Yan A (2012). Metabolic reprogramming under microaerobic and anaerobic conditions in bacteria. Subcell Biochem.

[CR40] Shao J, He Y, Chen A, Peng L, Luo S, Wu G, Zou H, Li R (2015). Interactive effects of algicidal efficiency of *Bacillus* sp. B50 and bacterial community on susceptibility of *Microcystis aeruginosa* with different growth rates. Int Biodeterior Biodegradation.

[CR41] Singh P (1974). Algicidal effect of 2,4-dichlorophenoxy acetic acid on blue–green alga *Cylindrospermum* sp. Arch Microbiol.

[CR42] Skerratt J, Bowman J, Hallegraeff G, James S, Nichols P (2002). Algicidal bacteria associated with blooms of a toxic dinoflagellate in a temperate Australian estuary. Mar Ecol Prog Ser.

[CR43] Su JF, Ma M, Wei L, Ma F, Lu JS, Shao SC (2016). Algicidal and denitrification characterization of *Acinetobacter* sp. J25 against *Microcystis aeruginosa* and microbial community in eutrophic landscape water. Mar Pollut Bull.

[CR44] Trotter EW, Rolfe MD, Hounslow AM, Craven CJ, Williamson MP, Sanguinetti G, Poole RK, Green J (2011). Reprogramming of *Escherichia coli* K-12 metabolism during the initial phase of transition from an anaerobic to a micro-aerobic environment. PLoS ONE.

[CR45] Wang X, Gong L, Liang S, Han X, Zhu C, Li Y (2005). Algicidal activity of rhamnolipid biosurfactants produced by *Pseudomonas aeruginosa*. Harmful Algae.

[CR46] Wang M, Bai J, Chen WN, Ching CB (2010). Metabolomic profiling of cellular responses to carvedilol enantiomers in vascular smooth muscle cells. PLoS ONE.

[CR47] Yamamoto Y, Kouchiwa T, Hodoki Y, Hotta K, Uchida H, Harada K-I (1998). Distribution and identification of actinomycetes lysing cyanobacteria in a eutrophic lake. J Appl Phycol.

[CR48] Yoshikawa K, Adachi K, Nishijima M, Takadera T, Tamaki S, K-i Harada, Mochida K, Sano H (2000). β-Cyanoalanine production by marine bacteria on cyanide-free medium and its specific inhibitory activity toward cyanobacteria. Appl Environ Microbiol.

[CR49] Yu X, Cai G, Wang H, Hu Z, Zheng W, Lei X, Zhu X, Chen Y, Chen Q, Din H (2018). Fast-growing algicidal *Streptomyces* sp. U3 and its potential in harmful algal bloom controls. J Hazard Mater.

[CR50] Zhang B, Cai G, Wang H, Li D, Yang X, An X, Zheng X, Tian Y, Zheng W, Zheng T (2014). *Streptomyces alboflavus* RPS and its novel and high algicidal activity against harmful algal bloom species *Phaeocystis globosa*. PLoS ONE.

[CR51] Zhao L, Chen L, Yin P (2014). Algicidal metabolites produced by *Bacillus* sp. strain B1 against *Phaeocystis globosa*. J Ind Microbiol Biotechnol.

