# Supplementary material for: Metabolomics analysis of Pseudomonas chlororaphis JK12 algicidal activity under aerobic and micro-aerobic culture condition
Source: AMB Express. 2018 Aug 20;8:131. doi: 10.1186/s13568-018-0660-x (PMC6102160; doi:10.1186/s13568-018-0660-x)
Supplement: Supplementary file 1 — Additional file 1. Fig. S1. ESI mass spectrum recorded by LC–MS analysis in positive ion mode for JK12 metabolites from a aerobic culture and b micro-aerobic culture. Fig. S2. ESI mass spectrum recorded by LC–MS analysis in negative ion mode for JK12 metabolites from a aerobic culture and b micro-aerobic culture. Fig. S3. PCA plot based on LC–MS analysis in positive ion mode for JK12 metabolites from aerobic and micro-aerobic culture. Fig. S4. PCA plot based on LC–MS analysis in negative ion mode for JK12 metabolites from aerobic and micro-aerobic culture. Fig. S5. Heatmap correlation based on LC–MS analysis in positive ion mode for JK12 metabolites from aerobic and micro-aerobic culture. Fig. S6. Heatmap correlation based on LC–MS analysis in negative ion mode for JK12 metabolites from aerobic and micro-aerobic culture. Table S1. List of identified metabolites extracted from JK12 media based on LC–MS analysis in positive ion mode. Table S2. List of identified metabolites extracted from JK12 media based on LC–MS analysis in negative ion mode. [file 13568_2018_660_MOESM1_ESM.docx]

**AMB Express**

**ADDITIONAL FILE**

**Metabolomics analysis of *Pseudomonas chlororaphis* JK12 Algicidal Activity Under Aerobic and Micro-aerobic Culture Condition**

Jaejung Kim^1^, Xiaomei Lyu^1^, Jaslyn Jie Lin Lee^1^, Guili Zhao^1^, Chin Seow Fong^2^, Liang Yang^2^*, Wei Ning Chen^1^*

* Corresponding authors:

Wei Ning Chen

Email: [WNChen@ntu.edu.sg](mailto:WNChen@ntu.edu.sg)

Tel: (+65) 6316 2870

Liang Yang

Email: [Yangliang@ntu.edu.sg](mailto:Yangliang@ntu.edu.sg)

Tel: (+65) 65923085

**^1^Address****:** School of Chemical and Biomedical Engineering, College of Engineering, Nanyang Technological University, 62 Nanyang Drive, Singapore 637459, Singapore

**Tel:** (+65)6316 2870

**^2^Address:** Singapore Centre for Environmental Life Sciences Engineering, Nanyang Technological University, 60 Nanyang Drive, Singapore 637551, Singapore

**List of Figures**

**Fig. S1** ESI mass spectrum recorded by LC-MS analysis in positive ion mode for JK12 metabolites from **a.** aerobic culture and **b.** micro-aerobic culture

**Fig. S2** ESI mass spectrum recorded by LC-MS analysis in negative ion mode for JK12 metabolites from **a.** aerobic culture and **b.** micro-aerobic culture

**Fig. S3** PCA plot based on LC-MS analysis in positive ion mode for JK12 metabolites from aerobic and micro-aerobic culture

**Fig. S4** PCA plot based on LC-MS analysis in negative ion mode for JK12 metabolites from aerobic and micro-aerobic culture

**Fig. S5** Heatmap correlation based on LC-MS analysis in positive ion mode for JK12 metabolites from aerobic and micro-aerobic culture

**Fig. S6** Heatmap correlation based on LC-MS analysis in negative ion mode for JK12 metabolites from aerobic and micro-aerobic culture

**List of Tables**

**Table S1** List of identified metabolites extracted from JK12 media based on LC-MS analysis in positive ion mode

**Table S2** List of identified metabolites extracted from JK12 media based on LC-MS analysis in negative ion mode

**
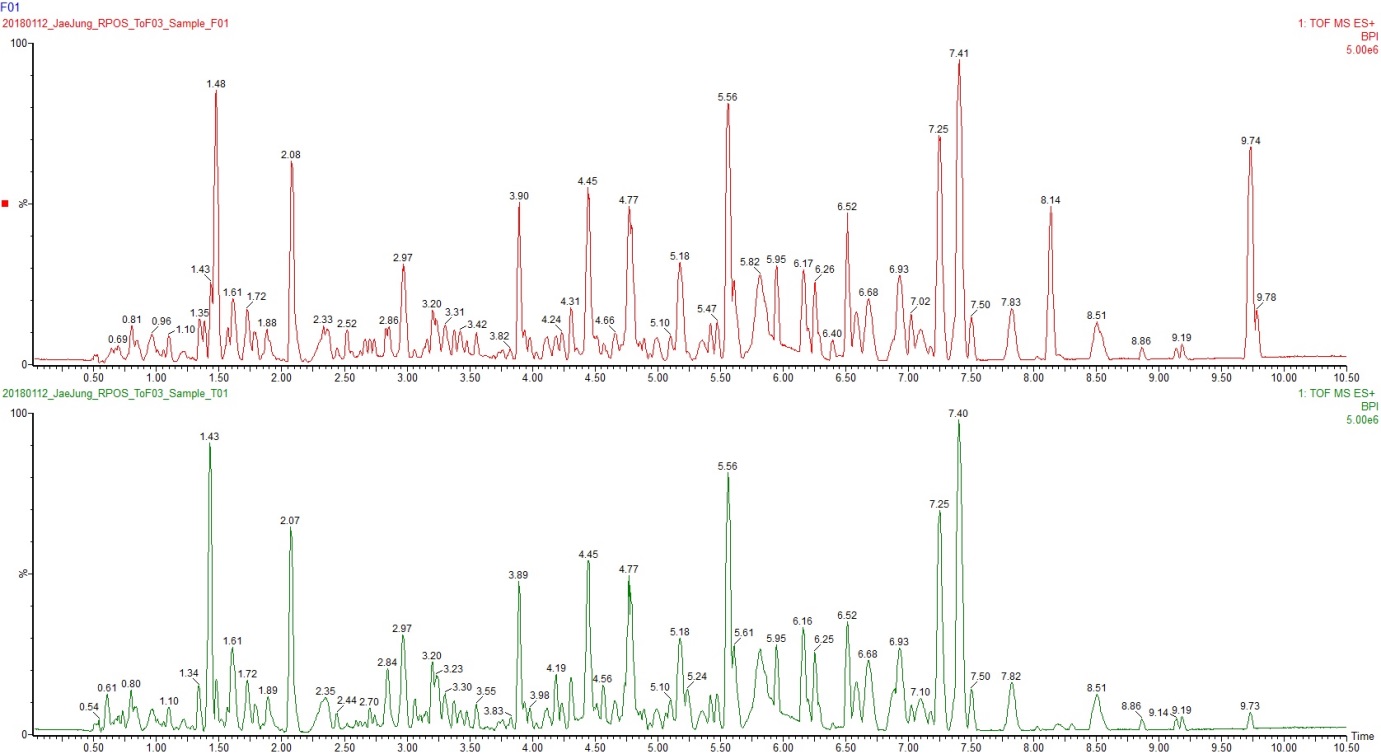
**

b

a

a

**Fig. S1** ESI mass spectrum recorded by LC-MS analysis in positive ion mode for JK12 metabolites from **a.** aerobic culture and **b.** micro-aerobic culture


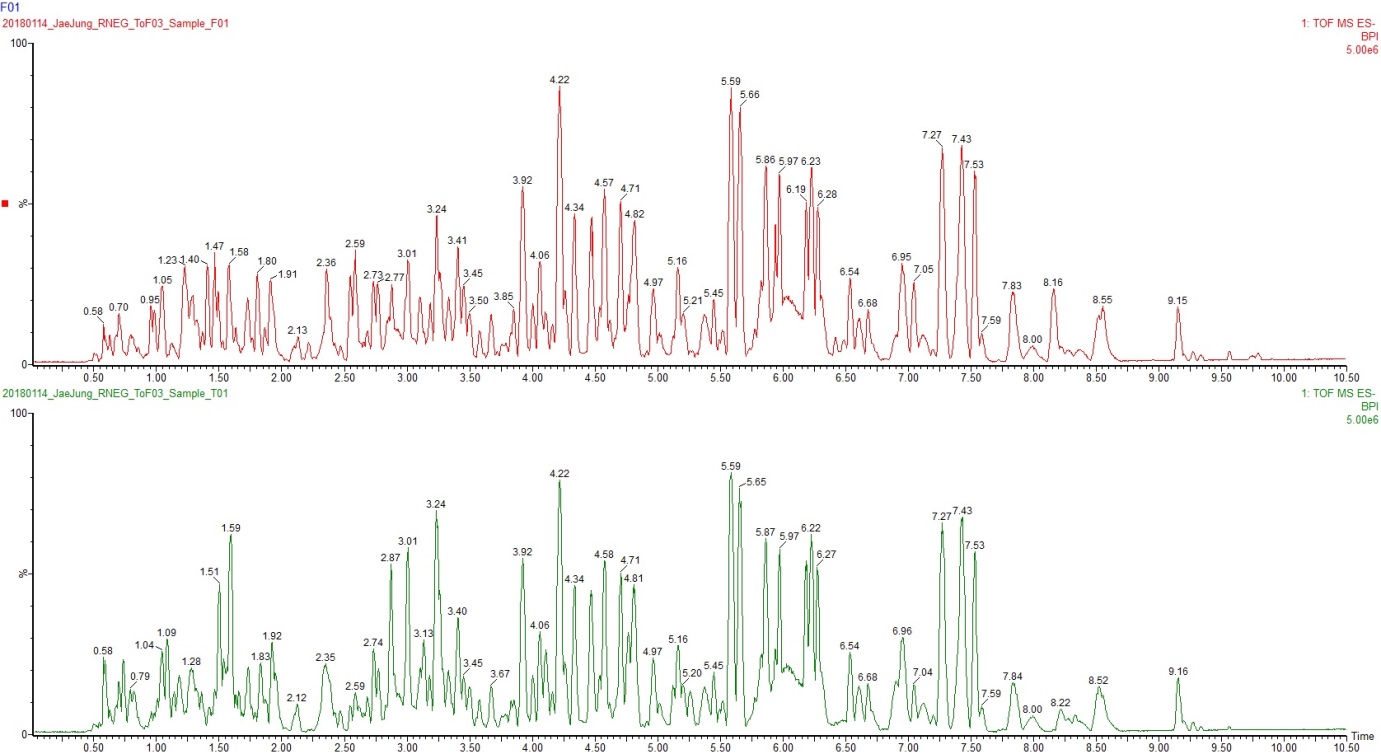


b

**Fig. S2** ESI mass spectrum recorded by LC-MS analysis in negative ion mode for JK12 metabolites from **a.** aerobic culture and **b.** micro-aerobic culture


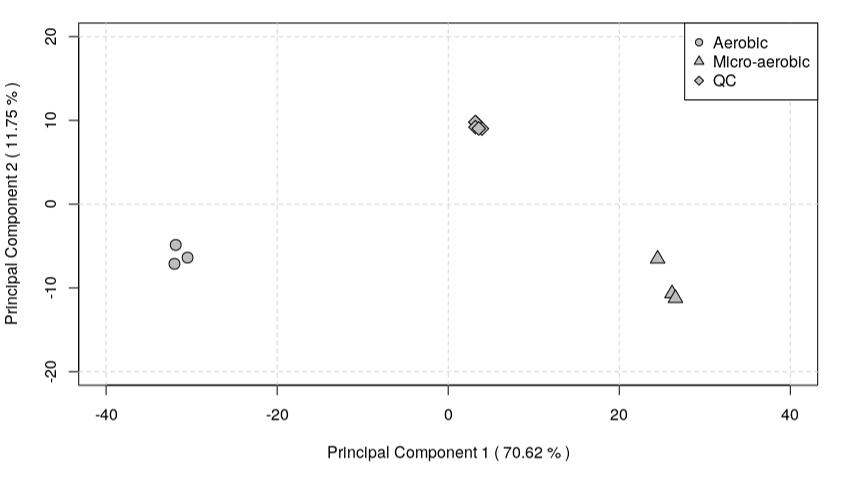


**Fig. S3** PCA plot based on LC-MS analysis in positive ion mode for JK12 metabolites from aerobic and micro-aerobic culture


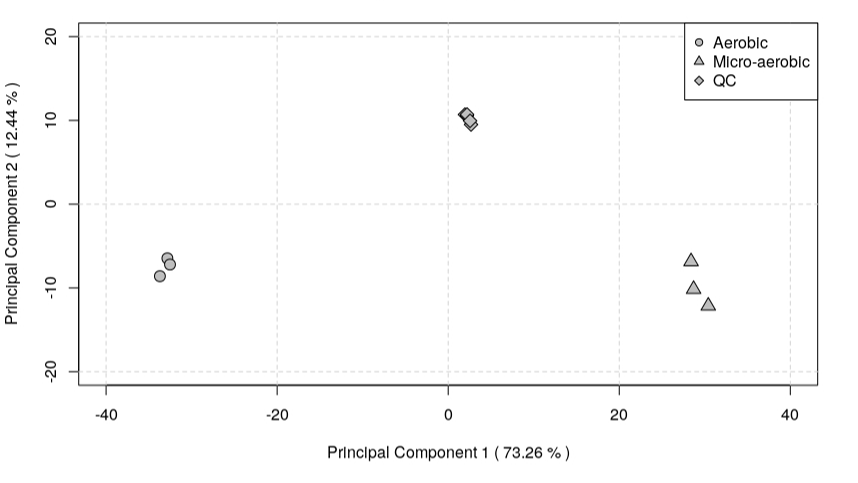


**Fig. S4** PCA plot based on LC-MS analysis in negative ion mode for JK12 metabolites from aerobic and micro-aerobic culture


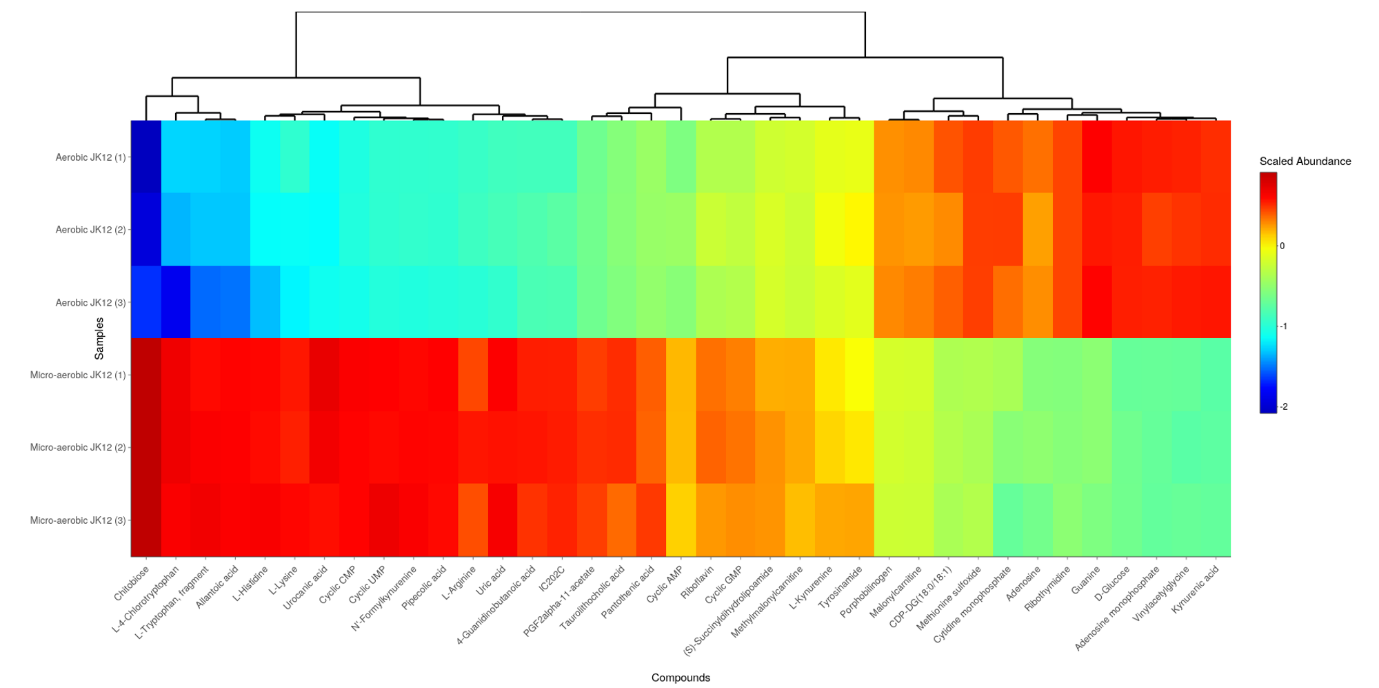


**Fig. S5** Heatmap correlation based on LC-MS analysis in positive ion mode for JK12 metabolites from aerobic and micro-aerobic culture


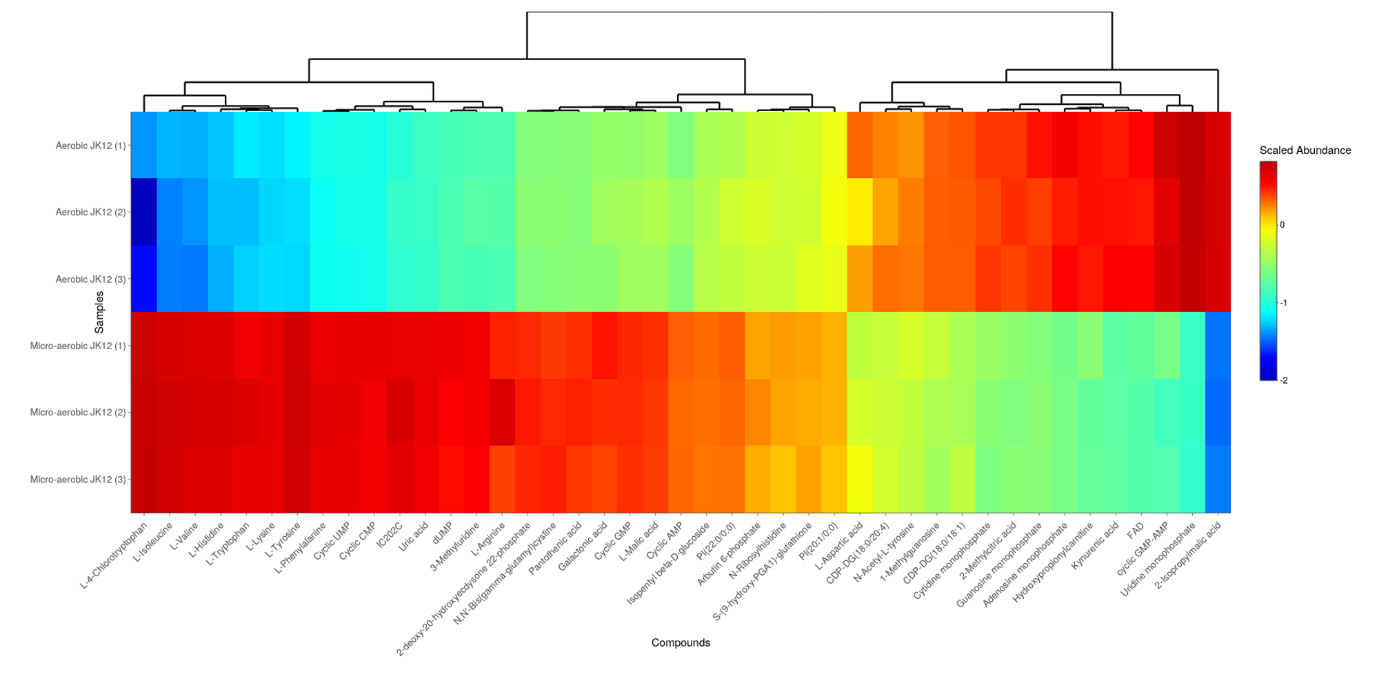


**Fig. S6** Heatmap correlation based on LC-MS analysis in negative ion mode for JK12 metabolites from aerobic and micro-aerobic culture

**Table S1** List of identified metabolites extracted from JK12 media based on LC-MS analysis in positive ion mode

| **Feature** | **ANOVA**  **p-value** | **Putative Identification** | | **Aerobic JK12** | | **Micro-aerobic JK12** | |
| --- | --- | --- | --- | --- | --- | --- | --- |
|  |  | **Name** | **ID** | **Intensity** | **%CV** | **Intensity** | **%CV** |
| 0.50_177.0616m/z | 8.22E-06 | Allantoic acid | HMDB01209 | 8,189.28 | 22.28 | 480,626.07 | 2.74 |
| 0.50_427.1525m/z | 2.05E-05 | Chitobiose | HMDB03556 | 365.74 | 62.13 | 967,011.34 | 0.50 |
| 0.57_147.1136m/z | 2.41E-05 | L-Lysine | HMDB00182 | 895.23 | 21.57 | 14,163.45 | 5.98 |
| 0.57_156.0773m/z | 1.10E-05 | L-Histidine | HMDB00177 | 5,340.43 | 19.12 | 142,197.82 | 5.77 |
| 0.57_175.1197m/z | 8.78E-06 | L-Arginine | HMDB00517 | 57,378.03 | 6.87 | 425,968.41 | 10.13 |
| 0.61_166.0540m/z | 2.05E-06 | Methionine sulfoxide | HMDB02005 | 276,086.31 | 0.13 | 148,856.34 | 2.58 |
| 0.64_248.1147m/z | 4.25E-05 | Malonylcarnitine | HMDB02095 | 429,449.56 | 1.95 | 338,679.61 | 0.86 |
| 0.66_144.0661m/z | 2.13E-06 | Vinylacetylglycine | HMDB00894 | 192,334.26 | 3.61 | 46,360.58 | 4.75 |
| 0.69_397.2562m/z | 3.82E-08 | PGF2alpha-11-acetate | 4266024 | 120,842.37 | 0.27 | 402,920.36 | 1.85 |
| 0.70_259.0935m/z | 4.07E-07 | Ribothymidine | HMDB00884 | 1,753,932.59 | 0.22 | 719,848.19 | 2.50 |
| 0.73_306.0498m/z | 1.17E-07 | Cyclic CMP | HMDB11691 | 146,359.71 | 5.02 | 2,127,122.98 | 2.04 |
| 0.92_130.0869m/z | 1.25E-07 | Pipecolic acid | HMDB00070 | 28,422.98 | 4.76 | 303,779.29 | 1.06 |
| 0.93_146.0930m/z | 6.16E-07 | 4-Guanidinobutanoic acid | HMDB03464 | 67,716.15 | 3.90 | 406,394.97 | 3.91 |
| 0.93_181.0705m/z | 3.95E-07 | D-Glucose | HMDB00122 | 179,131.77 | 1.24 | 47,316.69 | 3.46 |
| 0.95_324.0603m/z | 0.0005 | Cytidine monophosphate | HMDB00095 | 68,603.98 | 5.25 | 29,040.80 | 13.72 |
| 0.96_517.3349m/z | 1.64E-06 | IC202C | LMFA08020184 | 209,593.13 | 6.90 | 1,190,155.30 | 0.42 |
| 1.00_139.0508m/z | 1.54E-06 | Urocanic acid | HMDB00301 | 10,782.63 | 5.17 | 245,489.47 | 10.92 |
| 1.02_262.1297m/z | 4.04E-06 | Methylmalonylcarnitine | HMDB13133 | 1,132,116.04 | 0.80 | 1,338,901.07 | 0.23 |
| 1.07_307.0335m/z | 1.76E-06 | Cyclic UMP | HMDB11640 | 177,486.20 | 4.59 | 2,104,311.87 | 9.00 |
| 1.11_169.0363m/z | 2.46E-06 | Uric acid | HMDB00289 | 113,752.47 | 6.96 | 966,230.18 | 6.16 |
| 1.38_227.1036m/z | 2.41E-06 | Porphobilinogen | HMDB00245 | 653,301.46 | 0.73 | 535,010.77 | 0.48 |
| 1.38_348.0713m/z | 1.40E-06 | Adenosine monophosphate | HMDB00045 | 4,118,450.12 | 5.08 | 1,054,283.62 | 0.58 |
| 1.44_152.0571m/z | 1.81E-06 | Guanine | HMDB00132 | 1,396,388.43 | 3.47 | 438,235.52 | 3.12 |
| 1.47_330.0607m/z | 7.06E-05 | Cyclic AMP | HMDB11616 | 3,001,312.45 | 5.39 | 5,246,635.98 | 1.96 |
| 1.56_346.0557m/z | 1.23E-05 | Cyclic GMP | HMDB11629 | 2,064,230.53 | 2.17 | 3,004,501.02 | 1.20 |
| 1.71_268.1052m/z | 5.66E-05 | Adenosine | HMDB00050 | 185,245.43 | 4.98 | 89,295.52 | 4.96 |
| 1.78_308.0985m/z | 0.0001 | (S)-Succinyldihydrolipoamide | HMDB01177 | 370,875.25 | 1.04 | 451,249.95 | 1.94 |
| 2.41_237.0876m/z | 7.05E-07 | N'-Formylkynurenine | HMDB01200 | 23,645.69 | 6.57 | 261,104.54 | 3.85 |
| 2.65_209.0927m/z | 0.0135 | L-Kynurenine | HMDB00684 | 1,026,764.27 | 1.30 | 1,106,129.27 | 2.78 |
| 2.66_181.0975m/z | 0.1025 | Tyrosinamide | HMDB13319 | 111,885.05 | 1.86 | 117,809.50 | 3.81 |
| 2.73_220.1189m/z | 1.56E-05 | Pantothenic acid | HMDB00210 | 124,604.41 | 1.11 | 259,480.54 | 5.05 |
| 3.25_188.0713m/z | 1.67E-05 | L-Tryptophan, fragment | HMDB00929 | 181,073.22 | 25.44 | 11,002,632.68 | 9.14 |
| 3.69_190.0505m/z | 1.07E-06 | Kynurenic acid | HMDB00715 | 1,118,687.15 | 3.89 | 255,731.32 | 3.59 |
| 4.87_377.1462m/z | 0.0002 | Riboflavin | HMDB00244 | 277,841.86 | 4.18 | 411,445.79 | 3.11 |
| 5.07_239.0589m/z | 0.0003 | L-4-Chlorotryptophan | HMDB30400 | 2,990.77 | 54.16 | 344,169.70 | 5.35 |
| 5.51_484.3100m/z | 2.10E-05 | Taurolithocholic acid | 24702367 | 88,395.18 | 1.19 | 231,747.95 | 6.98 |
| 7.03_1008.5723m/z | 7.32E-05 | CDP-DG(18:0/18:1) | HMDB06979 | 10,479,453.09 | 4.64 | 6,565,398.58 | 1.05 |

**Table S2** List of identified metabolites extracted from JK12 media based on LC-MS analysis in negative ion mode

| **Feature** | **ANOVA**  **p-value** | **Putative Identification** | | **Aerobic JK12** | | **Micro-aerobic JK12** | |
| --- | --- | --- | --- | --- | --- | --- | --- |
|  |  | **Name** | **ID** | **Intensity** | **%CV** | **Intensity** | **%CV** |
| 0.55_145.0972m/z | 1.17E-09 | L-Lysine | HMDB00182 | 49,340.81 | 1.95 | 1,576,807.50 | 1.11 |
| 0.56_154.0611m/z | 5.24E-08 | L-Histidine | HMDB00177 | 17,457.06 | 4.85 | 848,780.75 | 4.32 |
| 0.57_173.1031m/z | 0.0001 | L-Arginine | HMDB00517 | 20,093.98 | 2.84 | 104,820.86 | 20.31 |
| 0.58_195.0501m/z | 1.21E-05 | Galactonic acid | HMDB00565 | 3,749,047.50 | 3.60 | 8,184,125.26 | 3.62 |
| 0.73_304.0331m/z | 4.80E-08 | Cyclic CMP | HMDB11691 | 487,053.05 | 0.80 | 6,946,763.27 | 4.32 |
| 0.76_132.0291m/z | 0.0292 | L-Aspartic acid | HMDB00191 | 30,113.32 | 6.18 | 25,722.73 | 5.36 |
| 0.86_116.0707m/z | 5.07E-07 | L-Valine | HMDB00883 | 10,877.08 | 12.81 | 833,783.90 | 0.77 |
| 0.88_307.0324m/z | 6.35E-07 | dUMP | HMDB01409 | 108,581.66 | 1.39 | 643,409.43 | 5.43 |
| 0.96_322.0436m/z | 1.05E-05 | Cytidine monophosphate | HMDB00095 | 6,256,084.84 | 2.26 | 2,759,785.52 | 4.65 |
| 0.97_515.3190m/z | 7.50E-07 | IC202C | LMFA08020184 | 92,998.79 | 4.69 | 1,024,342.75 | 6.26 |
| 1.09_305.0173m/z | 3.88E-08 | Cyclic UMP | HMDB11640 | 1,361,722.54 | 4.28 | 21,996,453.92 | 0.70 |
| 1.14_167.0201m/z | 4.58E-08 | Uric acid | HMDB00289 | 555,388.74 | 2.75 | 4,769,739.14 | 2.13 |
| 1.23_323.0278m/z | 6.19E-09 | Uridine monophosphate | HMDB00288 | 17,784,490.73 | 1.90 | 1,220,087.08 | 1.80 |
| 1.42_346.0551m/z | 2.72E-06 | Adenosine monophosphate | HMDB00045 | 10,706,538.80 | 5.08 | 3,199,751.00 | 1.85 |
| 1.47_180.0656m/z | 7.65E-08 | L-Tyrosine | HMDB00158 | 129,909.35 | 6.68 | 4,815,417.91 | 0.97 |
| 1.47_362.0500m/z | 5.76E-06 | Guanosine monophosphate | HMDB01397 | 10,105,873.15 | 4.76 | 4,020,543.96 | 1.62 |
| 1.49_286.1036m/z | 3.34E-05 | N-Ribosylhistidine | HMDB02089 | 1,349,471.44 | 0.44 | 1,568,558.27 | 1.19 |
| 1.50_328.0445m/z | 2.82E-05 | Cyclic AMP | HMDB11616 | 7,942,235.28 | 5.62 | 15,798,332.06 | 0.64 |
| 1.54_130.0864m/z | 8.79E-07 | L-Isoleucine | HMDB00172 | 19,776.44 | 14.64 | 1,683,753.17 | 5.23 |
| 1.59_133.0145m/z | 6.74E-06 | L-Malic acid | HMDB00156 | 120,363.18 | 3.58 | 225,641.20 | 0.20 |
| 1.59_344.0393m/z | 1.98E-06 | Cyclic GMP | HMDB11629 | 12,419,480.55 | 2.96 | 25,478,751.25 | 0.31 |
| 1.63_257.0771m/z | 6.38E-07 | 3-Methyluridine | HMDB04813 | 712,610.27 | 4.93 | 4,001,638.23 | 2.27 |
| 1.67_205.0344m/z | 2.84E-06 | 2-Methylcitric acid | HMDB00379 | 1,275,365.68 | 2.36 | 543,108.79 | 3.04 |
| 1.81_673.0924m/z | 3.45E-05 | cyclic GMP-AMP | HMDB60465 | 281,187.55 | 6.26 | 39,663.80 | 15.87 |
| 1.87_296.0983m/z | 3.66E-05 | 1-Methylguanosine | HMDB01563 | 277,803.99 | 0.23 | 176,730.60 | 3.94 |
| 2.23_497.1007m/z | 2.65E-06 | N,N'-Bis(gamma-glutamyl)cystine | HMDB38458 | 77,626.58 | 1.66 | 190,687.01 | 3.64 |
| 2.25_351.0480m/z | 0.0002 | Arbutin 6-phosphate | nnF5b@911lWF@lo | 212,968.72 | 1.16 | 249,252.88 | 1.80 |
| 2.35_164.0708m/z | 4.20E-08 | L-Phenylalanine | HMDB00159 | 390,532.37 | 3.46 | 6,912,646.72 | 2.98 |
| 2.36_249.1343m/z | 1.70E-06 | Isopentyl beta-D-glucoside | HMDB34750 | 155,728.03 | 1.52 | 229,156.72 | 0.28 |
| 2.76_218.1025m/z | 6.57E-07 | Pantothenic acid | HMDB00210 | 576,611.61 | 1.53 | 1,383,866.78 | 2.31 |
| 3.14_232.1180m/z | 2.59E-05 | Hydroxypropionylcarnitine | HMDB13125 | 440,723.98 | 1.13 | 128,211.32 | 9.99 |
| 3.27_203.0816m/z | 1.70E-06 | L-Tryptophan | HMDB00929 | 103,586.63 | 10.28 | 3,511,704.05 | 9.70 |
| 3.36_543.2717m/z | 4.11E-07 | 2-deoxy-20-hydroxyecdysone 22-phosphate | 85300615 | 1,867,155.79 | 1.75 | 4,835,710.88 | 2.02 |
| 3.48_644.3222m/z | 0.0002 | S-(9-hydroxy-PGA1)-glutathione | HMDB13059 | 424,803.89 | 1.40 | 479,696.64 | 0.84 |
| 3.59_222.0760m/z | 6.34E-05 | N-Acetyl-L-tyrosine | HMDB00866 | 145,592.94 | 1.66 | 112,903.44 | 1.90 |
| 3.72_188.0343m/z | 7.49E-07 | Kynurenic acid | HMDB00715 | 987,696.02 | 4.35 | 227,853.77 | 2.05 |
| 3.83_784.1497m/z | 6.60E-07 | FAD | HMDB01248 | 395,917.13 | 3.47 | 86,163.92 | 3.34 |
| 3.87_175.0600m/z | 9.94E-09 | 2-Isopropylmalic acid | HMDB00402 | 233,437.11 | 1.82 | 1,970.73 | 4.97 |
| 5.09_237.0427m/z | 0.0001 | L-4-Chlorotryptophan | HMDB30400 | 3,199.78 | 68.30 | 1,297,294.64 | 4.49 |
| 7.04_1006.5592m/z | 3.47E-05 | CDP-DG(18:0/18:1) | HMDB06979 | 18,597,878.19 | 0.74 | 11,589,660.00 | 4.01 |
| 7.04_1028.5408m/z | 0.0004 | CDP-DG(18:0/20:4) | HMDB06982 | 2,489,119.42 | 2.89 | 1,977,840.67 | 2.09 |
| 7.12_625.3352m/z | 0.0005 | PI(20:1/0:0) | 123066181 | 12,164,418.64 | 0.49 | 12,687,373.83 | 0.53 |
| 7.75_655.3813m/z | 0.0001 | PI(22:0/0:0) | 123066186 | 2,413,614.37 | 3.77 | 3,367,639.49 | 0.48 |
